# Comparison of manual and automated methods in the hematological evaluation of a frugivorous bat captured in urban green areas

**DOI:** 10.1007/s11259-026-11474-z

**Published:** 2026-10-02

**Authors:** Melissa Harumi Sumiyoshi, Eva Munyque da Cruz, Morgana Maira Hennig, Marcela Natacha Aparecida Rocha, Kayana Mota Caribé de Figueiredo, Nathália Soares Lima Rabelo, Gabriel de Mello, Glenda Akimi Ota, Guilherme Ratts de Almeida, Jéssica Inácio Gomes, Jhonathan França Bello, Mylene Karoline da Silva de Jesus, Alan Eriksson, Richard de Campos Pacheco, Rosa Helena dos Santos Ferraz, Adriane Jorge Mendonça

**Affiliations:** 1 Laboratory of Veterinary Clinical Pathology, Faculty of Veterinary Medicine, Veterinary Hospital, Federal University of Mato Grosso – UFMT, Cuiabá, Mato Grosso, Avenue Fernando Correa da Costa, Cuiabá, 2367, 78060-900 Brazil; 2 Graduate Program in Veterinary Sciences, Faculty of Veterinary Medicine, Federal University of Mato Grosso – UFMT, Mato Grosso, Avenue Fernando Correa da Costa, Cuiabá, 2367, 78060-900 Brazil; 3 Department of Biology and Zoology, Institute of Biosciences, Federal University de Mato Grosso – UFMT, Mato Grosso, Avenue Fernando Correa da Costa, Cuiabá, 2367, 78060-900 Brazil; 4 Marechal Rondon Faculty – FARON, Avenue Marechal Rondon, Vilhena, 10058, 76980-000 Rondônia Brazil

**Keywords:** *Artibeus planirostris*, Erythrogram, Leukogram, Neutrophil:lymphocyte ratio, Phyllostomidae

## Abstract

Increasing and unplanned urbanization can intensify wildlife contact with humans and domestic animals, raising concerns about the spillover of zoonotic pathogens. Bats play a relevant ecological role due to their great niche diversity and wide distribution, and may act as reservoirs for various pathogens. Despite their importance, hematological values for non-hematophagous bats remain limited, hindering the assessment of their health and physiological responses. This study characterized hematological parameters and blood cell morphology in *Artibeus planirostris* captured in urban green areas of Cuiabá, Brazil, and evaluated agreement between automated and manual hematological methods. Blood samples from 24 bats were analyzed using an automated hematology analyzer, microhematocrit, cyanmethemoglobin assay and blood smear microscopy for differential leukocyte counts, platelet estimation and morphological assessment. Erythrogram values were consistent with patterns previously reported in bats and may reflect physiological adaptations to flight metabolism. More than half of the individuals showed neutrophil predominance over lymphocytes; however, the influence of capture-related stress, infection and anesthetic effects could not be excluded. Bland-Altman and correlation analyses revealed variable agreement between automated and manual methodologies. Although some parameters showed good correlation, systematic biases and high variability indicated limited interchangeability between methods. These findings highlight the need for cautious interpretation of automated hematological analyses in bats and support the combined use of automated and manual approaches. Further studies with larger sample sizes are needed to improve method standardization and establish representative hematological reference data for bats in anthropized environments.

## Introduction

With increasing urbanization and environmental changes, wildlife faces habitat loss and closer contact with humans and domestic animals, which can lead to the emergence of zoonotic pathogens (Miguel et al. 2019; Tazerji et al. 2022). The order Chiroptera comprises a great diversity of species with morphophysiological variations and plays essential ecological roles, including seed dispersal, insect control and pollination (Strumpf et al. 2020). However, their ecological plasticity and adaptation to anthropogenic environments may also increase their exposure to stressors and pathogens (Villaba-Alemán et al. 2020). Therefore, understanding their physiology, ecology and epidemiology is important for One Health monitoring (Tazerji et al. 2022; Teles et al. 2023).

Hematological evaluation is valuable for assessing health and physiological status, allowing the identification of anemia, inflammatory and infectious responses, chronic conditions and dehydration associated with health disorders in animals (Miguel et al. 2019; Strumpf et al. 2020). Hematological monitoring may also contribute to environmental surveillance and zoonotic pathogens investigations (Hansen et al. 2022).

*Artibeus planirostris* is a Neotropical frugivorous bat of the family Phyllostomidae widely distributed throughout South America. Species of the genus *Artibeus* play important roles in seed dispersal and are highly adaptable to both natural and anthropized environments (Gardner 2007).

Despite the ecological and epidemiological importance of bats, hematological data are limited for non-hematophagous and synanthropic bats (Kuzel et al. 2020; Teles et al. 2023). In addition, few studies have characterized hematological parameters and blood cell morphology in *A. planirostris*, especially in urban environments (Miguel et al. 2019; Teles et al. 2023). Furthermore, the applicability and agreement of automated hematological analyzers for this species are still poorly understood, since species-specific blood characteristics may affect analytical accuracy (Hansen et al. 2022).

Due to the high frequency of *A. planirostris* captures in urban and peri-urban environments and its synanthropic occurrence, this study aimed to describe the hematological parameters and blood cell morphology of specimens captured in urban green areas of Cuiabá, Brazil. Additionally, it compared results from automated and manual/semi-automated methods to evaluate their agreement and applicability.

## Materials and methods

### Capture, identification and sample collection

All bats sampled in this study were collected during two nocturnal sampling events conducted in January and March 2025 at two locations in Cuiabá, Mato Grosso, Brazil. The region is predominantly characterized by the Cerrado biome, with a rainy season extending from October to March. Sampling was conducted at the Center for Medicine and Research in Wild Animals of the Federal University of Mato Grosso (UFMT) (15°36′26″S, 56°03′51″W), located within an urban matrix with fragmented vegetation patches, and at Mãe Bonifácia State Park (15°34′44″S, 56°05′16″W), an urban conservation area characterized by native Cerrado vegetation. A total of 44 bats were captured, of which 24 *A. planirostris* met the inclusion criteria. Samples showing clotting or fibrin formation and individuals from other species were excluded.

Bats were captured using mist nets in accordance with Ferreira et al. (2021), which were checked at 30-minute intervals. Captured bats were placed individually in cloth bags for approximately 20–30 min before handling and sample collection. Next, through physical examination and visual inspection, each animal was weighed, examined for ectoparasites, sexed and assessed for reproductive status, with pregnant or lactating animals released. Bats were anesthetized by intraperitoneal injection of xylazine hydrochloride (10 mg/kg) and ketamine hydrochloride (60 mg/kg), and at least 0.5 mL of blood samples were subsequently collected by cardiac puncture and stored into EDTA microtainer tubes. Following sedation, animals were euthanized as part of a broader research project requiring organ and tissue collection for complementary analyses, in accordance with ethical approvals. Species identification was performed using established taxonomic keys (Diaz et al. 2021; Gardner 2007).

### Sample processing

Samples were processed at the Veterinary Clinical Pathology Laboratory of UFMT within 2–12 h after collection due to field logistics associated with nocturnal capture, and kept refrigerated until analysis. Blood smears were prepared and stained using a Diff-quick stain (Laborclin^®^, Pinhais, Brazil). Hematocrit was determined using the microhematocrit technique by centrifugation of capillary tubes at 10,277 g for 5 min. Hemoglobin concentration was determined by the hemiglobin cyanide (CiCN) method using a commercial kit (Labtest^®^, Lagoa Santa, Brazil) and spectrophotometric reading at 540 nm (Bio 200, Bioplus^®^, São Paulo, Brazil) (Campbell and Grant 2022).

Hematological parameters, including total erythrocyte, leukocyte and platelet counts, hematocrit, hemoglobin concentration, Mean Corpuscular Volume (MCV) and Mean Corpuscular Hemoglobin Concentration (MCHC), were obtained using a pocH-100iV Diff automated hematology analyzer (Sysmex^®^, São José dos Pinhais, Brazil). The analyzer was operated in the “OTHER” research mode, with reference limits customized using hematological data reported for *Artibeus lituratus* by Kuzel et al. (2020). For each hematological parameter, the lower and upper limits were calculated as the reported mean minus one standard deviation and mean plus one standard deviation, respectively, and entered into the analyzer. No bat-specific calibration or correction factors were applied. The analyzer uses direct-current detection for erythrocyte, leukocyte and platelet counting, cyanide-free photometry for hemoglobin determination and cumulative pulse-height detection for hematocrit measurement. Although used in wildlife studies, this analyzer has not been validated for bats and, to our knowledge, has not previously been evaluated in this group.

Differential leukocyte counts generated by the analyzer were presented in three categories for comparison with the manual method: small white blood cells, assumed to correspond to lymphocytes according to the manufacturer; middle white blood cells, assumed to correspond to neutrophils, monocytes and basophils; and large white blood cells, assumed to correspond to eosinophils, although this classification may be limited in non-domestic species.

Total erythrocyte, leukocyte and platelet counts were not performed manually due to the volume of samples processed simultaneously, as priority was given to timely sample handling in order to minimize delays between collection and analysis.

### Microscopic examination

Blood smears were examined using a light microscope (Leica DM 500, Leica Microsystems, Heerbrugg, Switzerland) to assess cell morphology and to perform manual differential leukocyte counts, based on 100 cells per slide, at 1000× magnification with oil immersion. Absolute leukocyte counts for each cell type were subsequently calculated using the total leukocyte count obtained from the automated hematology analyzer. All slides were independently evaluated by two observers. Platelet counts were estimated by averaging the number of platelets in ten random fields within the monolayer at the same magnification, and the mean value was multiplied by a factor of 15,000 (Stockham and Scott 2025).

### Statistical analysis

Data were analyzed using descriptive statistics in Microsoft Excel^®^, including mean, standard deviation and 90% confidence intervals. Given the limited sample size and the descriptive nature of the study, confidence intervals were included to aid interpretation of data variability. Neutrophil: lymphocyte (N: L) ratios were calculated based on differential leukocyte counts obtained by microscopic blood smear evaluation.

Comparisons between automated and manual methods were performed using nine paired hematological parameters. Data normality was assessed using the Shapiro-Wilk test in Jamovi software (version 2.6; The Jamovi Project, 2024). Pearson’s correlation coefficient was applied to normally distributed variables (hemoglobin and hematocrit), whereas Spearman’s correlation coefficient was used for the remaining parameters. Correlation strength was classified according to Bauer et al. (2011) as excellent (rs = 0.93–0.99), good (rs = 0.80–0.92), fair (rs = 0.59–0.79) or poor (rs < 0.59). Agreement between methods was evaluated by Bland-Altman analysis using MedCalc software (version 23.5.2; MedCalc Software Ltd., Ostend, Belgium), including calculation of bias and 95% limits of agreement. P-values from all correlation analyses were adjusted for multiple comparisons using the Benjamini-Hochberg procedure (Benjamini and Hochberg 1995) to control the false discovery rate, as implemented in R (version 4.4; R Core Team, 2024).

## Results

Hematological parameters of 24 specimens of *A. planirostris* are presented in Table 1. Due to the limited sample size and the exclusion of pregnant and lactating females, as well as the unbalanced distribution between sampling sites and periods, statistical analyses for reproductive status, age, season, and site comparisons were not performed. Of the 24 individuals, 16.7% (4/24) were females and 83.3% (20/24) were males, while 16.7% (4/24) were juveniles and 83.3% (20/24) were adults.

**Table 1:** Hematological parameters of *Artibeus planirostris* obtained by automated (a), semi-automated (sa) and manual (m) methods in urban areas of Cuiabá, Brazil

Parameters (Unit of measurement)	*n*	Mean (SD)	Min-Max Values	CI (90%)	Method
Erythrocytes (10^6^ /µL)	24	11.98 (1.47)	8.28–14.24	11.49–12.48	a
Hemoglobin (g/dL)	24	17.63 (1.60)	14.20–20.40	17.09–18.17	a
^1^ Hemoglobin (g/dL)	24	21.38 (2.73)	15.13–25.53	20.46–22.30	sa
Hematocrit (%)	24	59.40 (6.19)	45.90–70.40	57.30–61.40	a
^2^ Hematocrit (%)	24	52.50 (4.32)	44.00–60.00	51.00–54.00	m
MCV (fL)	24	49.70 (2.72)	45.70–56.60	48.80–50.60	a
MCHC (g/dL)	24	29.70 (0.72)	28.30–30.90	29.50–30.00	a
Leukocytes (10³/µL)	24	6.50 (3.74)	1.30–13.80	5.20–7.70	a
Small white blood cell (%)	24	71.00 (12.77)	55.40–93.30	66.70-75.28	a
Middle white blood cell (%)	24	14.00 (6.77)	3.70–23.60	11.72–16.27	a
Large white blood cell (%)	24	15.00 (6.67)	3.00–28.00	12.77–17.25	a
Small white blood cell (10³/µL)	24	4.80 (3.27)	0.90–12.00	3.65–5.85	a
Middle white blood cell (10³/µL)	24	0.80 (0.54)	0.10-2.00	0.62–0.98	a
Large white blood cell (10³/µL)	24	0.90 (0.65)	0.20–2.40	0.69–1.12	a
Band neutrophils (%)	24	0.00 (0.00)	0.00	0.00	m
Segmented neutrophils (%)	24	51.50 (19.99)	12.00–76.00	44.70–58.20	m
Eosinophils (%)	24	2.30 (2.99)	0.00–13.00	1.30–3.30	m
Basophils (%)	24	0.40 (1.24)	0.00–6.00	0,00-0.80	m
Lymphocytes (%)	24	42.80 (19.43)	20.00–84.00	36.30–49.40	m
Monocytes (%)	24	3.00 (2.52)	0.00–11.00	2.20–3.80	m
Band neutrophils (10³/µL)	24	0.00 (0.00)	0.00	0.00	m
Segmented neutrophils (10³/µL)	24	3.00 (1.86)	0.62–6.92	2.40–3.70	m
Eosinophils (10³/µL)	24	0.10 (0.19)	0.00-0.73	0.10–0.20	m
Basophils (10³/µL)	24	0.00 (0.03)	0.00-0.11	0.00–0.00	m
Lymphocytes (10³/µL)	24	3.10 (2.85)	0.48–10.50	2.10-4.00	m
Monocytes (10³/µL)	24	0.20 (0.25)	0.00-0.89	0.10–0.30	m
Platelets (10³/µL)	18	864.30 (231.87)	435.00-1326.00	779.00-949.60	a
^3^ Platelets (10³/µL)	18	500.30 (119.93)	232.00-725.00	456.20-544.40	m
Total plasma protein (g/dL)	23	5.90 (0.61)	5.00-7.20	5.70–6.10	m

*SD* Standard Deviation

^1^ Hemiglobin cyanide method. ^2^ microhematocrit method. ^3^ estimated in blood smears

**Fig. 1:**
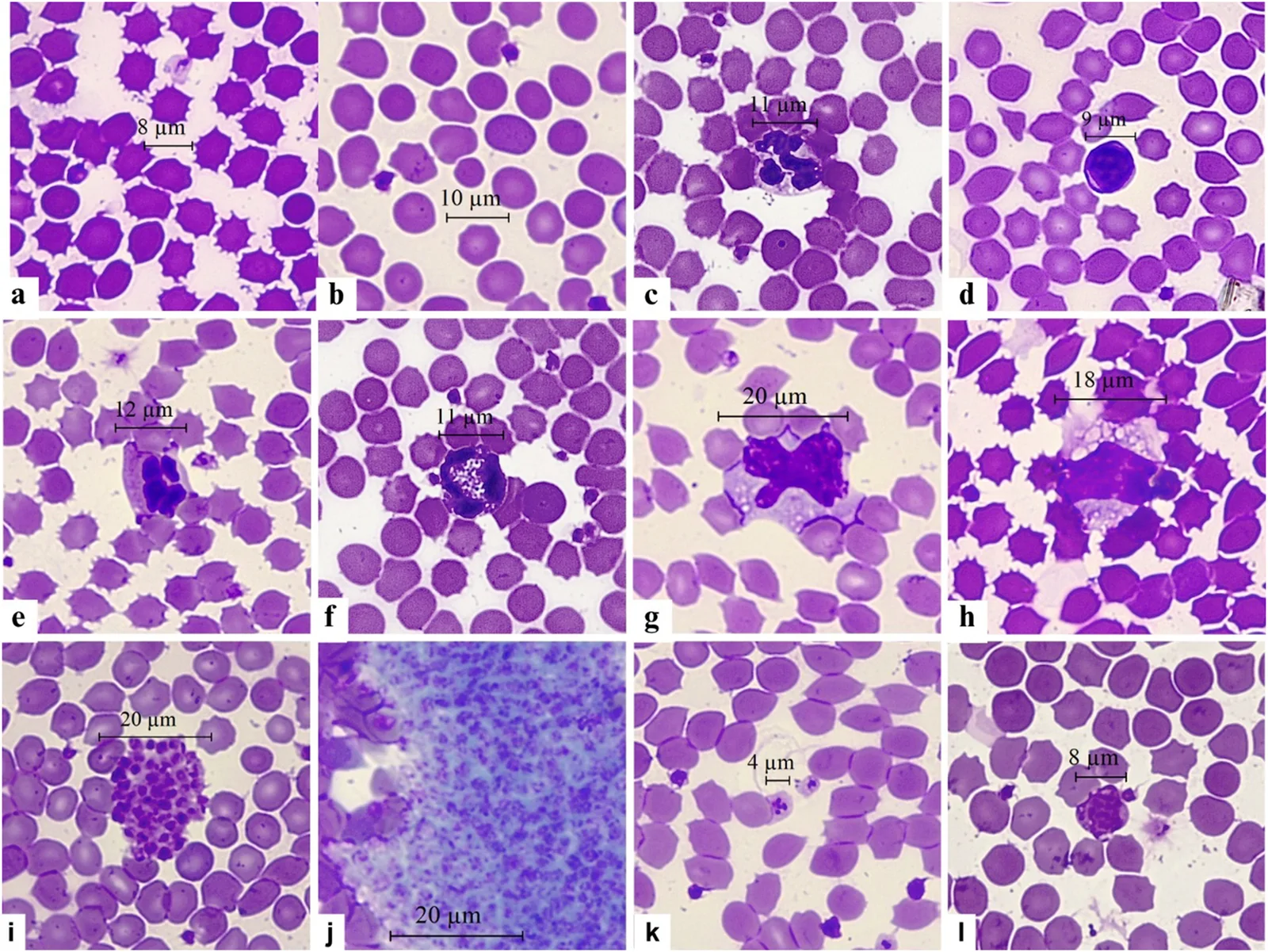
Photomicrographs of erythrocytes, leukocytes and platelets morphology in blood smears of *Artibeus planirostris* stained with Diff-quick and observed at 1000× magnification. Images acquired using a Leica DM500 microscope equipped with a digital camera and Leica Application Suite (LAS, Leica Microsystems). (**a**) echinocytes, (**b**) anisocytosis, (**c**) neutrophil, (**d**) lymphocyte, (**e**) eosinophil, (**f**) basophil, (**g**) monocyte, (h) activated monocyte, (**i-j**) platelet clumps, (**k**) basophilic inclusions in platelets, (**l**) macroplatelet

Erythrocytes exhibited normal morphology, appearing predominantly normocytic and normochromic. However, echinocytes (Fig. 1a) were observed in 41.6% (10/24) of individuals. One individual presented lower erythrocyte count, hemoglobin concentration, and hematocrit values compared to the remaining animals (8.28 × 10⁶/µL, 14.20 g/dL, and 45.90%, respectively, as measured by the pocH-100iV Diff analyzer). Hemoglobin and hematocrit values obtained by alternative methods were 18.36 g/dL (cyanmethemoglobin method) and 44.00% (microhematocrit technique), respectively. This individual also exhibited anisocytosis (Fig. 1b), which may represent individual variation.

Neutrophils (Fig. 1c) were the most frequent leukocyte type in 58.3% (14/24) of the sampled individuals, followed by lymphocytes (Fig. 1d) in 41.7% (10/24). Neutrophils presented agranular or hypogranular cytoplasm and dense nuclear chromatin, while lymphocytes were characterized by small size, high nucleus/cytoplasm ratio and a rounded to oval nucleus. The mean N: L ratio was 1.68 ± 0.24 (SD), with values ranging from 0.14 to 3.81.

Other leukocytes, including eosinophils (Fig. 1e), basophils (Fig. 1f) and monocytes (Fig. 1g) were presented in lower numbers. The eosinophils had small discrete pink granules and the basophils had more evident basophilic granules. Monocytes vary in color and size both among different individuals and within the same individual. The cytoplasm was blue to grayish in color, the chromatin was lighter when compared to the neutrophil, and the nucleus was irregularly shaped. Activated monocytes (Fig. 1h) were observed in 25% (6/24) of individuals and showed variable degrees of cytoplasmic vacuoles.

Platelets counts were excluded in six individuals due to moderate to intense platelet aggregation (Fig. 1i-j). Additionally, platelet inclusions (Fig. 1k) and macroplatelets (Fig. 1l) were observed in one individual. Total plasma protein could not be measured due to hemolysis in one sample.

Pearson’s correlation analysis demonstrated fair and good agreement between methods for hemoglobin (*r* = 0.728, *p* < 0.001) and hematocrit (*r* = 0.813, *p* < 0.001), respectively. Spearman’s correlation analysis was used for the remaining parameters. Absolute lymphocyte counts showed the strongest correlation (rs = 0.948, *p* < 0.001), followed by lymphocyte percentage (rs = 0.835, *p* < 0.001), the percentage of “others” leukocytes (neutrophils, monocytes, and basophils) (rs = 0.817, *p* < 0.001), and the absolute counts of “others” leukocytes (rs = 0.829, *p* < 0.001). Platelet counts showed a fair correlation (rs = 0.609, *p* = 0.002). In contrast, eosinophil percentage (rs = − 0.130, *p* = 0.546) and absolute eosinophil count (rs = 0.075, *p* = 0.727) were not significantly correlated between methods, suggesting that the analyzer may not reliably identify this leukocyte population in bats. After adjustment for multiple comparisons using the Benjamini-Hochberg procedure, all seven previously significant correlations remained significant (all adjusted *p* < 0.003), whereas eosinophil percentage and absolute eosinophil counts remained non-significant (adjusted *p* = 0.614 and 0.727, respectively), further supporting the robustness of these findings.

**Fig. 2:**
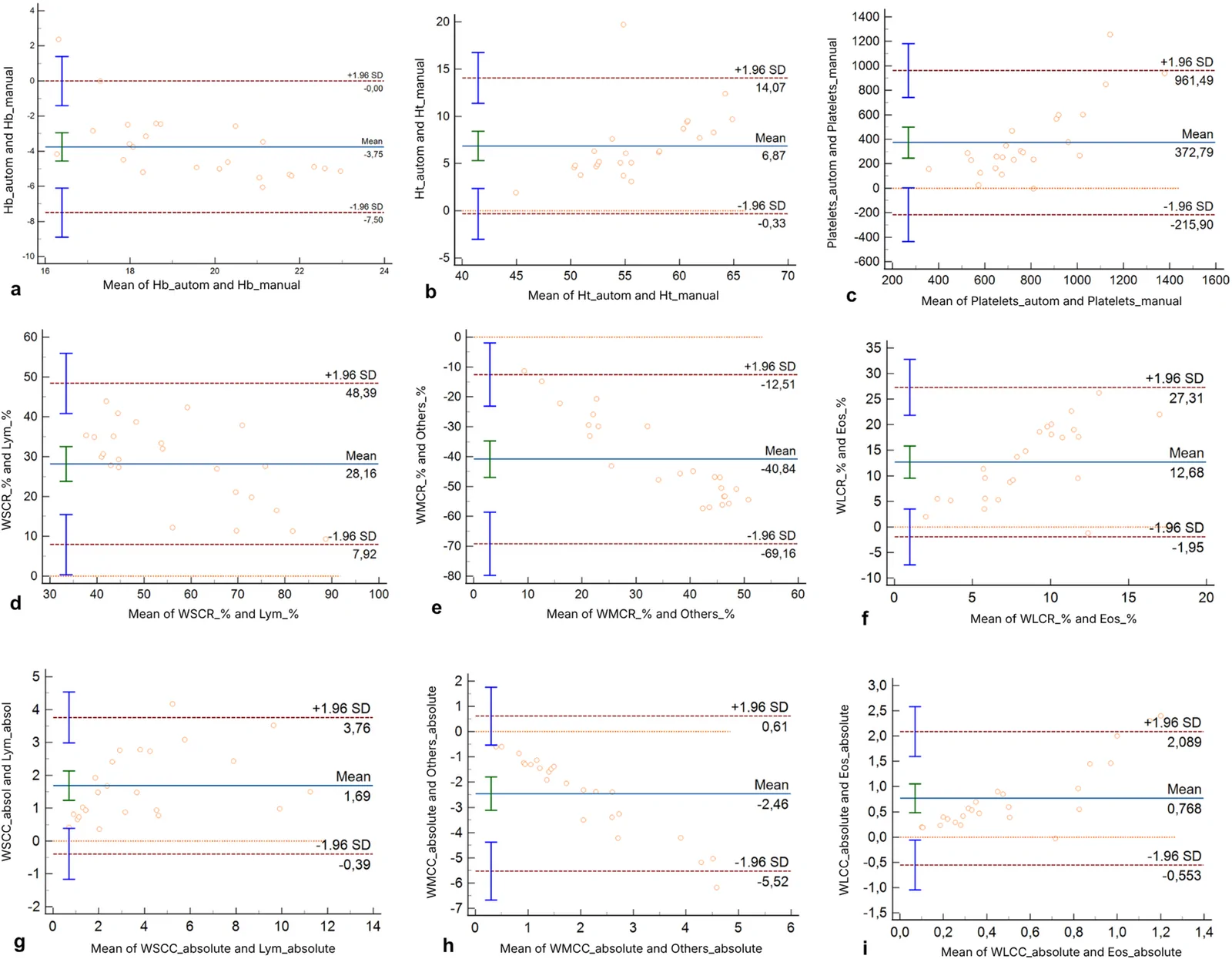
Agreement between manual and automated analyzer (pocH-100iV Diff, Sysmex) results for hemoglobin, hematocrit, platelets and leucocyte differential counts for *Artibeus planirostris*. Bland-Altman diagrams for hemoglobin (Hb) (**a**), hematocrit (Ht) (**b**), platelets (**c**), white small cell ratio (WSCR) and lymphocyte ratio (Lym) (**d**), white middle cell ratio (WMCR) and others (neutrophil, monocyte and basophil) ratio (**e**), white large cell ratio (WLCR) and eosinophil (Eos) ratio (**f**), white small cell count (WSCC) and lymphocyte count (Lym) (**g**), white middle cell count (WMCC) and others (neutrophil, monocyte and basophil) count (**h**), white large cell count (WLCC) and eosinophil (Eos) count (**i**). The difference between methods is plotted against the mean of both methods. The solid blue line represents the mean difference (bias) and the dotted red line the ± 1.96 standard deviation

Bland-Altman analyses demonstrated variable agreement between automated and manual methods (Fig. 2). Hemoglobin showed a negative bias (-3.75 g/dL), indicating lower values obtained by the automated analyzer, whereas hematocrit showed a positive bias (+ 6.87%). Platelet counts presented a marked positive bias (+ 372.8 × 10³/µL) and wide limits of agreement, indicating high variability between methods. Lymphocyte percentages also showed positive bias, while “others” leukocyte percentages demonstrated consistent negative bias. Eosinophil counts presented wide limits of agreement and poor concordance between methods. In contrast, absolute lymphocyte counts showed narrower limits of agreement and better agreement between methods.

## Discussion

This study provides a comparative evaluation of hematological parameters obtained by automated and manual methods in *A. planirostris* from urban green areas in central Brazil. Overall, variable agreement was observed between methodologies depending on the hematological parameter evaluated, while blood cell morphology and leukocyte profiles were generally consistent with previous reports in bats (Hansen et al. 2022; Kuzel et al. 2020; Teles et al. 2023).

Hematocrit, hemoglobin and erythrocyte values observed in the evaluated *A. planirostris* individuals are consistent with hematological patterns previously described in bats and may reflect physiological adaptations associated with the metabolic demands of sustained flight (Schinnerl et al. 2011; Miguel et al. 2019; Villalba-Alemán et al. 2020; Kuzel et al. 2020). However, these findings should be interpreted cautiously, since factors such as dehydration or physiological variations may also influence erythrogram parameters.

Echinocytes were observed in 41.6% of individuals, a morphological alteration previously reported in the pteropodid fruit bat *Pteropus alecto* (Hansen et al. 2022). In the present study, this finding was possibly associated with preparation artifacts, EDTA excess, dehydration, or stress (Thrall et al. 2024). One individual showed lower erythrogram values and anisocytosis, suggesting anemia, as previously described in other bat species (Schinnerl et al. 2011; Hansen et al. 2022).

As presented in Table 1, leukocyte analysis revealed neutrophil percentages higher than lymphocyte percentages in more than half of the individuals, resulting in N: L ratios above 1. Similar leukocyte patterns have been reported in other bat species, including *Artibeus* spp. (Hansen et al. 2022; Kuzel et al. 2020; Teles et al. 2023). This leukocyte pattern may be associated with stress responses related to capture and handling (Stockham and Scott 2025). The N: L ratio has been used as an indirect indicator of stress (Viola et al. 2022). Miguel et al. (2019) reported higher N: L ratios in frugivorous bats, including *A. lituratus* and *A. planirostris*, in areas with lower habitat availability.

Although lymphocyte predominance is commonly reported in many bat species (Schinnerl et al. 2011; Strumpf et al. 2020; Teles et al. 2023; Villalba-Alemán and Muñoz-Romo 2016; Villalba-Alemán et al. 2020), neutrophil predominance was more frequently observed in the present study. This finding may reflect differences in capture and handling procedures, anesthetic protocols, environmental conditions, the species or populations evaluated, or other methodological variations among studies. Xylazine and ketamine, the anesthetic drugs used in this study, may increase total leukocyte counts and induce leukogram changes similar to stress responses during anesthesia (Hanif et al. 2025). In bats, information regarding the effects of anesthetics on hematological parameters remains scarce, although increased lymphocyte counts following isoflurane anesthesia have been reported (Strumpf et al. 2020).

Monocytes showed typical morphology, with activated forms observed in some individuals, as previously reported in other bats (Hansen et al. 2022). These findings may occur in small numbers in domestic animals under normal conditions, but may also indicate inflammatory or infectious processes (Stockham and Scott 2025).

Basophilic inclusions in platelets compatible with *Anaplasma* sp. were observed in one individual, similar to previous reports in urban bats (Villalba-Alemán et al. 2020). However, confirmatory diagnosis requires molecular testing due to their similarity to dye precipitates or nuclear fragments (Stockham and Scott 2025). Macroplatelets were also identified in the same animal. Although their clinical significance in bats is unknown, in dogs they are associated with increased platelet regeneration, whereas in cats they may occur as a physiological finding (Thrall et al. 2024).

Bland-Altman and correlation analyses demonstrated variable agreement between automated and manual methodologies. Although good correlations were observed for hematocrit and lymphocyte counts, correlation alone does not indicate interchangeability between methods, since systematic biases were also identified. These findings suggest that the automated analyzer may detect relative variations in some hematological parameters while still producing substantially different absolute values.

Hemoglobin concentrations obtained by the automated analyzer were consistently lower than those obtained by the CiCN method, whereas hematocrit values showed positive bias. These discrepancies may reflect methodological differences between techniques, species-specific erythrocyte characteristics, and pre-analytical factors such as sample handling and time to analysis. Since the analyzer used was originally developed for domestic mammals, including dogs, cats, horses and cattle (Riond et al. 2011), analytical performance may have been affected by chiropterans’ hematological characteristics.

Platelet and eosinophil counts showed poor agreement and high variability between methods. Platelet discrepancies may be associated with aggregation and interference in impedance-based systems, whereas the low concordance observed for eosinophils suggests limitations in leukocyte differentiation in bats. Microscopic evaluation indicated that cells classified as large white blood cells by the analyzer frequently correspond to monocytes, suggesting possible leukocyte misclassification. Likewise, the automated leukocyte differential is not directly comparable with the microscopic N: L ratio, as the analyzer groups neutrophils, monocytes and basophils into a single cell category rather than identifying neutrophils separately. Together, these findings reinforce that automated hematological analyses in chiropterans should be interpreted cautiously and preferably combined with blood smear evaluation.

This study has limitations that should be considered when interpreting the results. The relatively small sample size, combined with the predominance of male individuals, may have influenced the hematological findings, particularly because pregnant and lactating females were released during field activities. Additionally, the short sampling period, as well as potential effects of anesthesia and sample processing time due to field logistics associated with nocturnal capture, may have contributed to hematological variability. The absence of manual hemocytometer counts and use of smear-based platelet estimation also constitutes methodological limitations of the present study. Finally, analytical limitations of the automated analyzer may also have influenced the results.

Nevertheless, this study provides the first hematological description of frugivorous bats from the state of Mato Grosso, Brazil, and contributes baseline data for future investigations. The comparison between methods demonstrates that automated hematology analyzers should be used with caution in bats, and combined automated and manual approaches are recommended. Further studies with larger sample sizes and longer sampling periods are needed to allow robust sex-related analyses, standardize automated techniques and establish more consistent and representative hematological parameters for bats in anthropized environments.

## Data Availability

No datasets were generated or analysed during the current study.
