# Correction: Comparison of manual and automated methods in the hematological evaluation of a frugivorous bat captured in urban green areas

**DOI:** 10.1007/s11259-026-11578-6

**Published:** 2026-10-17

**Authors:** Melissa Harumi Sumiyoshi, Eva Munyque da Cruz, Morgana Maira Hennig, Marcela Natacha Aparecida Rocha, Kayana Mota Caribé de Figueiredo, Nathália Soares Lima Rabelo, Gabriel de Mello, Glenda Akimi Ota, Guilherme Ratts de Almeida, Jéssica Inácio Gomes, Jhonathan França Bello, Mylene Karoline da Silva de Jesus, Alan Eriksson, Richard de Campos Pacheco, Rosa Helena dos Santos Ferraz, Adriane Jorge Mendonça

**Affiliations:** 1 Laboratory of Veterinary Clinical Pathology, Faculty of Veterinary Medicine, Veterinary Hospital, Federal University of Mato Grosso – UFMT, Avenue Fernando Correa da Costa, Cuiabá 2367, Cuiabá, 78060-900 Mato Grosso Brazil; 2 Graduate Program in Veterinary Sciences, Faculty of Veterinary Medicine, Federal University of Mato Grosso – UFMT, Mato Grosso, Avenue Fernando Correa da Costa, Cuiabá, 2367, 78060-900 Brazil; 3 Department of Biology and Zoology, Institute of Biosciences, Federal University de Mato Grosso – UFMT, Mato Grosso, Avenue Fernando Correa da Costa, Cuiabá, 2367, 78060-900 Brazil; 4 Marechal Rondon Faculty – FARON, Avenue Marechal Rondon, Vilhena, 10058, 76980-000 Rondônia Brazil

**Correction:Veterinary Research Communications.**

**https://doi.org/10.1007/s11259-026-11474-z**

In this article the wrong figure appeared as Fig. 1.; the figure should have appeared as shown below.

**Figure:**
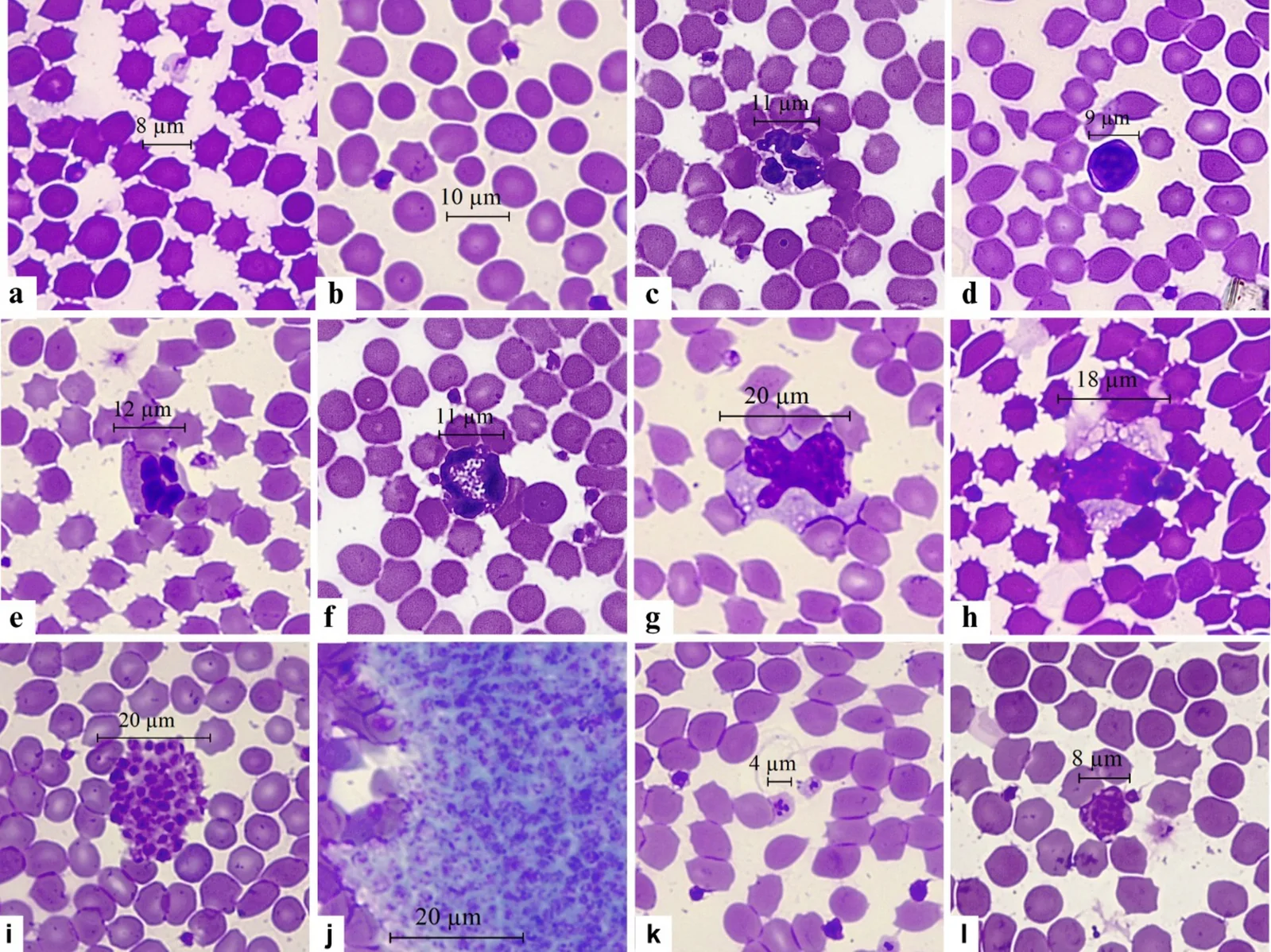

The original article has been corrected.

